# ‘You feel how you look’: Exploring the impacts of unmet water, sanitation, and hygiene needs among rural people experiencing homelessness and their intersection with drug use

**DOI:** 10.1371/journal.pwat.0000019

**Published:** 2022-05-25

**Authors:** April M. Ballard, Hannah L. F. Cooper, April M. Young, Bethany A. Caruso

**Affiliations:** 1 Gangarosa Department of Environmental Health, Emory University Rollins School of Public Health, Atlanta, GA, United States of America; 2 Department of Behavioral, Social, and Health Education Sciences, Emory University Rollins School of Public Health, Atlanta, GA, United States of America; 3 Department of Epidemiology, University of Kentucky College of Public Health, Lexington, Kentucky, United States of America; 4 Center on Drug and Alcohol Research, Department of Behavioral Science, University of Kentucky College of Medicine, Lexington, Kentucky, United States of America; 5 Hubert Department of Global Health, Emory University Rollins School of Public Health, Atlanta, GA, United States of America

## Abstract

Existing literature attests to water, sanitation, and hygiene (WASH) inequities among people experiencing homelessness (PEH) in the United States, but there is a dearth of research on such issues in rural areas. Homelessness is an emerging public health concern in rural areas where homelessness is on the rise, infectious disease outbreaks are becoming increasingly common, and PEH face unique WASH-related challenges compared to their urban counterparts. We conducted an exploratory study to understand the impacts of unmet WASH needs among rural PEH and their intersection with drug use through in-depth interviews (n = 10). Eligible participants were 18 years or older, lived in one of five Central Appalachian counties, and had experienced homelessness in the previous six months. Using thematic analysis, we identified factors that inhibit WASH access, and adverse health and well-being outcomes that result from unmet WASH needs. We also explore how WASH experiences compare among rural PEH who self-reported drug use to those who did not. Our findings revealed that factors at multiple levels inhibited WASH access, including stigma and place-based characteristics, which contributed to the adverse physical, mental, and emotional health of PEH. Comparisons between PEH who used drugs to those that did not revealed the intricate relationship between WASH, homelessness, and substance use in communities impacted by the opioid epidemic. Expanded WASH facilities that are safe and available with no prerequisites can address inadequate access among rural PEH and collaboration with harm reduction services may be advantageous to reach those who inject drugs.

## 1. Introduction

Water, sanitation, and hygiene (WASH) are critical to health and well-being [1–5], particularly among people experiencing homelessness (PEH) whose access to WASH is pivotal to preventing infectious disease outbreaks. The rapid transmission of SARS-CoV-2 [6–9] and widespread Hepatitis A (HAV) outbreak [10] in the United States (US) demonstrate the need for increased WASH access among PEH. Notably, the multistate HAV outbreak that began in 2016 resulted in over 30,000 cases across 32 states with experiencing homelessness emerging as a key risk factor for contracting the fecal-oral transmitted virus [10, 11]. As 2.3 to 3.5 million people experience homelessness in the US each year [12, 13], and economic conditions during the COVID-19 pandemic have likely exponentially increased the number of PEH, the availability of and access to WASH is critical for the nation’s health.

A robust portfolio of research demonstrates how homelessness adversely impacts health [14–24], while another documents the critical role that WASH plays in health [25–27]. Yet, few studies have explored the intersection of homelessness and WASH. US-based homelessness research has investigated various health outcomes related to WASH (e.g., reproductive health outcomes such as *Trichomonas vaginalis infection*, HAV infection), but the primary research focus has been on topics such as access to and acceptance of care [18–20], vaccination [21, 24], social determinants of health [28], and high-risk behaviors such as injection drug use 24] among others. Similarly, numerous studies have been conducted to show barriers to WASH such as the cost for infrastructure and resources [29–32] and social marginalization/ stigma [29, 30], and the physical, wellbeing, and psychosocial consequences of inadequate WASH [2, 4, 26, 27, 33], though these have been primarily from low- and middle-income settings. Global WASH research has been conducted among those who have been displaced or lack stable housing (e.g., residents of informal settlements) [30, 34, 35], but we were only able to identify three international WASH-focused studies on PEH [36–38].

The World Health Organization and United Nations Children’s Fund Joint Monitoring Programme (JMP) reports that in 2020, 2 billion people globally lack access to safely managed water services, defined as water services available on household premises, available when needed, and free from contamination. Additionally, 3.6 billion people globally lacked access to safely managed sanitation services, defined as facilities that are not shared with other households and that eventually have excreta treated on- or off-site. Lastly, 2.3 billion people lacked basic handwashing services in 2020, meaning they did not have a handwashing facility with soap and water at home [39]. However, these global estimates are based on data collected at the household level and therefore, by design, do not assess WASH access for PEH. Programmatic standards and monitoring guidance do exist for those experiencing housing insecurity and living in refugee settings, for example due to conflict or natural disaster [40], but no global guidance or strategy exists for assessing WASH access among those experiencing homelessness and not living in a setting established for those displaced.

Various known and unknown barriers may play a role in PEH’s reduced or lack of WASH access [11, 41–44]. Accessible public toilets, sinks, water, showers, and laundry facilities may be limited in number or absent due to the current sociocultural and political context in the US, including ‘toilet policies’ that reduce availability of and accessibility to public toilets such as having to pay for facility entry [45] and criminalization of homelessness [46, 47]. A study in Fresno, California found that PEH in encampments were urinating and defecating in public because they were denied access to facilities in local businesses and there were no public restrooms nearby. PEH also had difficulty finding somewhere to shower and reported no drinking water fountains nearby [48]. Similarly, a study in Detroit, Michigan found that PEH reported significant difficulties accessing public shower facilities; reported barriers included few facilities, concerns about thieves, long wait times, inconvenient hours, and privacy concerns [11]. In addition, a New York City-based study found that people living in shelters struggled to meet their menstrual health needs, because of lack of clean, functioning, and private toilets, and supplies like toilet paper [49].

To date, US-based WASH research is quite limited, and studies among PEH even more so. We note that the vast majority of existing research highlights WASH challenges ***urban*** PEH face in the US [11, 41, 42, 48, 49]. However, scant information exists about the unique WASH experiences of rural PEH. Homelessness is often perceived as an urban issue, yet rural homelessness does exist and is rising [50]. Paucity of jobs, economic disparities, and poor public housing infrastructure are structural drivers of rural homelessness [51]. In areas such as Appalachia that have been disproportionately impacted by the opioid epidemic [52–55], homelessness is likely exacerbated by substance use, which increases individual-level risk of homelessness [51, 56, 57]. Of particular concern is inadequate WASH access among people who inject drugs (PWID): difficulties cleaning the skin or washing hands before injection and use of unclean water for mixing or diluting drugs increases risk of bacterial infection and abscesses at injection sites [58–62].

This exploratory study fills an important gap in the literature around WASH inequities at the overlooked intersections of rurality, homelessness, and drug use. We sought to qualitatively investigate WASH experiences and the impacts of unmet WASH needs among PEH in rural Kentucky where resources are limited [63, 64] and research suggests homelessness may be prevalent, particularly among those who use drugs [65, 66]. We also explore how WASH experiences compare among rural PEH who self-reported drug use to those who did not.

## 2. Methods

### 2.1. Setting

This study was conducted in five counties in rural Central Appalachian Kentucky, whose names have not been included in order to protect the confidentiality of participants. The study area, like much of the region, struggles with poverty, inadequate healthcare, insufficient housing, and limited resources. Four of the five study counties are designated as distressed [64] and medically underserved [63]. The opioid epidemic and drug-related harms (e.g., overdose) have greatly impacted the study area, interacting with and exacerbating existing health, economic, and social disparities [67]. The population is overwhelmingly white (*>*90%) and most are high school graduates (77%) [68]. According to the Kentucky Housing Corporation’s (KHC) Point-in-Time Count (2019), three of the study counties have no PEH and a total of 30 PEH reside in the two remaining counties [69]. This appears to be an under-estimate, which has been routinely shown in Point-in-Time Counts [70] and as evidenced by a recent study among people who use drugs (PWUD) across the five counties that revealed 36.1% (n = 100) had experienced homelessness in the six months prior. The number of PEH in the study among PWUD alone exceeded the KHC estimate [66]. Two shelters (45 beds total) service the entire five-county area: one long-term shelter (i.e., on-site residence for up to 90 days) for adult men and women, and one emergency shelter for survivors of domestic violence. Shelters often have drug-free policies and previous convictions make some ineligible for public housing and other federal housing assistance.

### 2.2. Sample and participant selection

Participants were eligible if they were 18 years or older, lived in any of the five study counties, and had experienced homelessness in the previous six months. Homelessness was defined as living in a car, park, abandoned building, tent, squat, shelter, at a campsite, on the street, place-to-place, or “couch-surfing.” We used purposive quota sampling ensuring equal gender representation. Given the exploratory nature of this research, saturation was not prioritized. Initial participants were selected with the help of community researchers using a longitudinal cohort study database of people who had reported experiencing homelessness in the previous six months and agreed to be contacted for future research. Additionally, we gave a local shelter information about the study to share with residents. Interested residents contacted study staff to be screened for eligibility. Participants could also refer their peers.

### 2.3. Data collection

A woman (author initials removed for blinding) in her late twenties from a Kentucky county outside of the study area conducted in-depth interviews (IDIs) in March 2019. IDIs lasted approximately one hour and were audio recorded, transcribed verbatim, and de-identified. Basic demographics were collected via a short survey. Participants received $25 US cash for participating.

IDIs queried everyday WASH experiences; facilitators and barriers to WASH resources and facilities; and impacts of WASH experiences. Participants were asked to respond to questions in the context of when they were experiencing homelessness. This self-defined approach allowed participants to define what *they* consider homelessness, rather than researchers operationalizing the term. WASH components were explored separately, beginning with water, followed by hygiene and sanitation. Menstruators were asked about their experiences managing menses. If participants disclosed current or prior drug use, its relationship with homelessness and WASH was explored. The semi-structured interview guide is included in S1 Text.

### 2.4. Data analysis

We conducted thematic analysis. A codebook was developed using the interview guide, transcript readings, debriefing notes, and annotations. Transcripts were coded two at a time followed by memoing and review of the codebook. After coding, memos were written for each transcript, synthesizing and describing major themes. Segments from transcripts were queried by code and intersections of prominent codes across all transcripts, and thematic memos were developed. This process was repeated to compare themes across PEH who did and did not disclose drug use. We explored the impacts of both sex and gender during analyses given the unique WASH needs based on one’s biological sex and unique gender-related social experiences. Queries and memoing were used iteratively to conceptualize and describe key themes. We used Maslow’s Hierarchy of Needs [71] and the social ecological model [72–74] as sensitizing constructs, or concepts that inform research and “offer ways of seeing, organizing, and understanding experience” [75, p. 259]. Maslow’s Hierarchy of Needs is a psychological motivational theory wherein individuals need to have their lower needs (i.e., physiological and safety and security needs) met before they can attend to higher-level needs (i.e., psychological and self-fulfillment needs).[71] While we use the Hierarchy of Needs to organize our findings, we acknowledge that there is debate regarding the rigid order of needs and ethnocentricity of Maslow’s approach [76–78]. As such, we use the psychological theory as an opportunity to gain a wider perspective on WASH impacts on human health while also recognizing that human needs generally and hierarchically may vary at the individual level and across communities and cultures.

### 2.5. Ethics

All participants provided informed, verbal consent prior to data collection and received a copy of the consent form. If the participant verbally consented to participate, a paper oral consent form was signed by the study staff member conducting the informed consent discussion. A waiver of signature was requested and approved because confidentiality was a concern due to criminalization of homelessness and drug use and the study was otherwise minimal risk. Participants were made aware of their right to skip any question and end interviews at any time. The Emory University Institutional Review Board approved all study procedures.

## 3. Results

We identified perceived adverse health and well-being outcomes that result from unmet WASH needs, and factors that inhibit access to WASH resources and facilities. We also compare outcomes and WASH experiences among rural PEH who reported drug use to those who did not. We first present critical context from the data that illuminates factors that influence types of rural homelessness and what WASH resources, facilities, and behaviors PEH use when attempting to meet their needs.

### 3.1. Rural homelessness and WASH

The final sample included ten non-Hispanic white participants, six cisgender women and four cisgender men, aged 28 to 54 (mean = 38, Interquartile range [IQR]: 36.5, 41.5). On average, participants had lived in their residential county for 29 years, with eight having lived there for 15 years or more. Four participants (2 women, 2 men) self-disclosed substance use during interviews. Participants reported experiencing multiple ‘types’ of homelessness over time, oscillating between places and utilizing a variety of resources, facilities, and behaviors to meet their WASH needs as their sheltering experiences shifted. Substance use was critical to this discourse.

#### 3.1.1. Factors that influence types of rural homelessness.

We identified four main types of homelessness: (1) living place-to-place or “couch-surfing,” (2) living outdoors or in cars, (3) living in structures without water and/or electricity, and (4) living in a shelter for PEH or survivors of domestic violence. Every participant reported sleeping at a social network member’s place during the previous six months (type 1). Most had slept at parks (80%) (type 2), while only two participants had stayed at a shelter (20%) (type 4). Participants also slept in hospital waiting areas, motels, baseball dugouts, graveyards, barns, and public bathrooms (types 1, 2 and 3), though these were less common. All participants moved between various types of homelessness. For example, within a six-month period, one man (age 28) lived in his car, but stayed with friends when it was cold and had recently moved to a shelter.

Various factors influenced the types of homelessness experienced (Table 1). Many participants with strong connections and/or numerous family members and friends were able to move between places or “couch-surf.” Participants stayed with people for short periods, making sure not to “overstay their welcome.” Season also influenced dependence on others:

“Yeah, now that it’s warm, there’s going to be a lot more [PEH]. They’re not staying with just whoever. They’re not making do in bad situations where they can break free and try to get in a better situation, like removing themselves from some type of abuse that they went ahead and dealt with...when the weather is warmer, they’ll be less likely to take abuse and things that are going on and try to do different” **(woman, age 35)**

Some participants lived outdoors or in cars when they lacked social support, and knew many others who had too, mainly during warmer months. When participants lived outdoors, they stayed out of sight in the woods near natural water resources (e.g., lakes).

Three participants had lived in structures that had no running water and/or electricity (e.g., mobile homes), which they described as experiencing homelessness.

“Well even with the people that do have some type of shelter that they’ve made or are staying in during the dry time, even having the rain water...That’s what you use to do your cleaning, your dishes, and even your bathroom duties, to keep things clean in your area. Whether it is in a home or in a dwelling that has been made.” (**woman, age 35**)

Lack of water and/or electricity was due to a lack of public water infrastructure or inability to pay bills.

Three participants discussed living in a shelter, and two were living at one at the time of the interview. Most of the remaining participants were unwilling or unable to live at shelters because of limited capacity; lack of knowledge about how shelters function; or requirements to complete background checks, have photo identification, or have no felony charges. Participants had different understandings of requirements. Most believed that shelters required people to have no felony charges while one participant explained, “you can have felonies, drug charges and stuff like that. You just can’t have no violent crimes,” (man, age 40). Participants who did and did not use drugs explained how substance use was a barrier to shelters, due to lack of privacy, required on-site residency, and policies banning substance use.

Homelessness was described as more prevalent recently because of increasing substance use in the area. When asked why the number of PEH was increasing, one participant explained:

“Definitely drugs. ‘Cause every one of them that I know is homeless is either on drugs now or trying to get off drugs...they spend every penny and every waking moment of their time trying to chase that drug so it will take over your life. That’s exactly what happened to me. I lost my home, my house, my car, everything over it.” **(man, age 40)**

The interplay between substance use and homelessness emerged in most interviews despite not being directly queried; it was woven throughout four participants’ experiences. Those who did and did not use drugs described substance use as a catalyst for homelessness that led to and perpetuated poverty, and influenced the ‘type’ of homelessness experienced. For example, current and previous substance use strained or limited social network ties PEH who used drugs could rely on, leading them to “beg” and compromise their well-being and comfort or go without shelter. Conversely, PEH who did not use drugs with strong or numerous social network members could live place-to-place or “couch-surf.”

The realities of homelessness also influenced substance use for three participants who used drugs. Participants reported how homelessness lowered their self-esteem and self-worth, leading them to want to use drugs more and escape reality through an altered state of consciousness.

“...it wouldn’t be asking [to stay with someone]. I’d have to basically beg, and it’s bad. I guess that’s why I used so much. ‘Cause then I didn’t care...the harder it was, the more I didn’t want to be conscious.” **(woman, age 27)**

#### 3.1.2. WASH resources, facilities, and behaviors.

Water, sanitation, and hygiene were interconnected and rarely considered in isolation. Participants described sanitation facilities as critical for meeting water and hygiene needs.

##### 3.1.2.1. Water.

Drinking water sources included: (1) purchased water (e.g., bottled); (2) natural sources (e.g., springs); (3) public faucets at parks; (4) neighbors, friends, and family; (5) water fountains or sinks at gas stations, hospitals, and convenience stores; and (6) outdoor church pantries (called “blessing boxes”). Water for other purposes (e.g., bathing) was retrieved from bathrooms or facilities in public spaces, social network members’ houses, and natural sources. Participants strategically rationed and recycled their water.

“You can always put bleach in your rainwater and use it to clean your place, you can use it to flush with, you can use it for a lot of different things...you can actually recycle your water and you learn to conserve that and use what you have because, you know, it’s limited.” **(woman, age 35)**

Season, location, and perceived water quality influenced which sources were leveraged and for what purpose. During warmer months, participants were more likely to live outdoors and utilize natural sources. Participants made judgements about water quality and use based on the source. Creek or rainwater were mostly used for hygiene, cooking, or flushing toilets lacking running water. Purchased water or water from public faucets was used for drinking. Participants also drank water from natural sources, and would boil it depending on the source. Some consumed spring water without boiling it because it was “naturally filtered.”

##### 3.1.2.2. Sanitation.

PEH urinated and defecated outdoors, in public restrooms, flush toilets without running water, and buckets. A majority reported urinating (80%) and defecating (60%) outdoors and had used a bucket or flush toilet with no running water (60%) in the previous six months. Public restrooms included facilities at parks (e.g., portable restrooms), restaurants, convenience stores, and gas stations. One participant relied on their social network’s bathrooms while others found it embarrassing to ask to use someone’s bathroom.

Facilities alone were insufficient for meeting sanitation-related needs. Toilet paper or something comparable was also required. Traveling with toilet paper or toilet paper substitutes was necessary due to being transient and using various sanitation locations. Most relied on free napkins at gas stations or restaurants and baby wipes. Some used clothing.

“...it’s even kind of embarrassing...trying to get napkins and things from gas stations...that’s really the only access that you have...It’s hard...Sometimes you just make do with other things you might find available...If you have old socks that have holes in them, you’re going to use that instead.” **(woman, age 35)**

Type of sanitation facility used depended on type of homelessness, location, transportation access, and facility characteristics. Those who were transient (e.g., those who lived place-to-place or “couch-surfed”) relied on public facilities, while those who were living in a structure utilized buckets or flush toilets without water more frequently. Being closer to town was advantageous while being further limited facility access, making outdoor urination and defecation more likely. Participants with functioning vehicles could access a greater variety of facilities.

Facility characteristics influenced which public facilities PEH used, including requirements for patronage prior to use and the physical and social environment of establishments. Participants leveraged their extensive knowledge of facilities’ layouts and access procedures (e.g., asking for a key) to determine which to use.

“...there’s an Arby’s in the middle of town, you don’t even have to walk in the store. They have a walk-in door and then there’s the bathroom, then there’s another door...but like... [other places] you have to walk all the way into the store to get to the bathroom...”**(woman, age 27)**

##### 3.1.2.3. Hygiene.

Hygiene was important and complex. PEH emphasized numerous resources needed to manage, maintain, and meet hygiene needs. Body, hair, hand, fingernail, dental, and clothing hygiene were described, though body, hair, and clothing were most prominent. Only women mentioned hair as a hygiene component. Menstruation was discussed separately.

Participants needed water, products (e.g., shampoo), facilities, washing machines, and privacy to manage hygiene adequately. Table 2 outlines hygiene management strategies participants utilized. Most relied on public facilities and social network members. Personal hygiene products were acquired through public facilities, social network members, social service organizations, churches, and purchasing. Toilet paper, soap, and paper towels were acquired from convenience stores and restaurant and gas station bathrooms. One participant relied entirely on one social service organization for products and facilities.

Participants used the same facilities for hygiene that they used for water and sanitation, deciding where to perform hygiene behaviors based on facility characteristics.

“... whenever you’re in a public bathroom or a place like a Kroger where you have a family bathroom where you can lock the door, that’s where you want to try to maybe wash in the sink, wash your hair and your body. Try to change whatever clothing you may have.” **(woman, age 35)**

Menstruation was an added challenge; many menstruators had irregular periods. Menstruation required participants to wash their body and clothes more frequently, requiring extra soap and water. They relied on their housed social network members more during menstruation. One participant used baby wipes and soap together in the absence of water to clean herself during menstruation.

“I would keep the big packs of [baby wipes] for when I didn’t have a whole lot of water or in case we ran out...I would put soap on those, like actual soap, body wash or whatever, and then rinse them back out and rinse myself back off. That was about the only thing you could do.” **(woman, age 42)**

Participants used tampons, pads, and panty liners to catch blood. In their absence, they used “toilet paper, uh rags, sock. Just whatever you had” **(woman, age 54)**. One participant made tampons out of toilet paper.

### 3.2. WASH barriers and adverse outcomes

Factors at multiple levels inhibited WASH access, resulting in unmet basic (i.e., physiological and safety and security needs) and psychological needs (i.e., belongingness and love and esteem needs). We describe the adverse health and well-being impacts experienced by participants, aligned with Maslow’s Hierarchy of Needs. Then, we describe factors that serve as WASH barriers for PEH using the social ecological model. Throughout, we compare and contrast barriers and adverse outcomes among those who did and did not use drugs. Adverse outcomes and barriers for each WASH element largely overlapped and are therefore discussed jointly.

#### 3.2.1. Adverse impacts on basic and psychological health of PEH.

**Fig 1.**
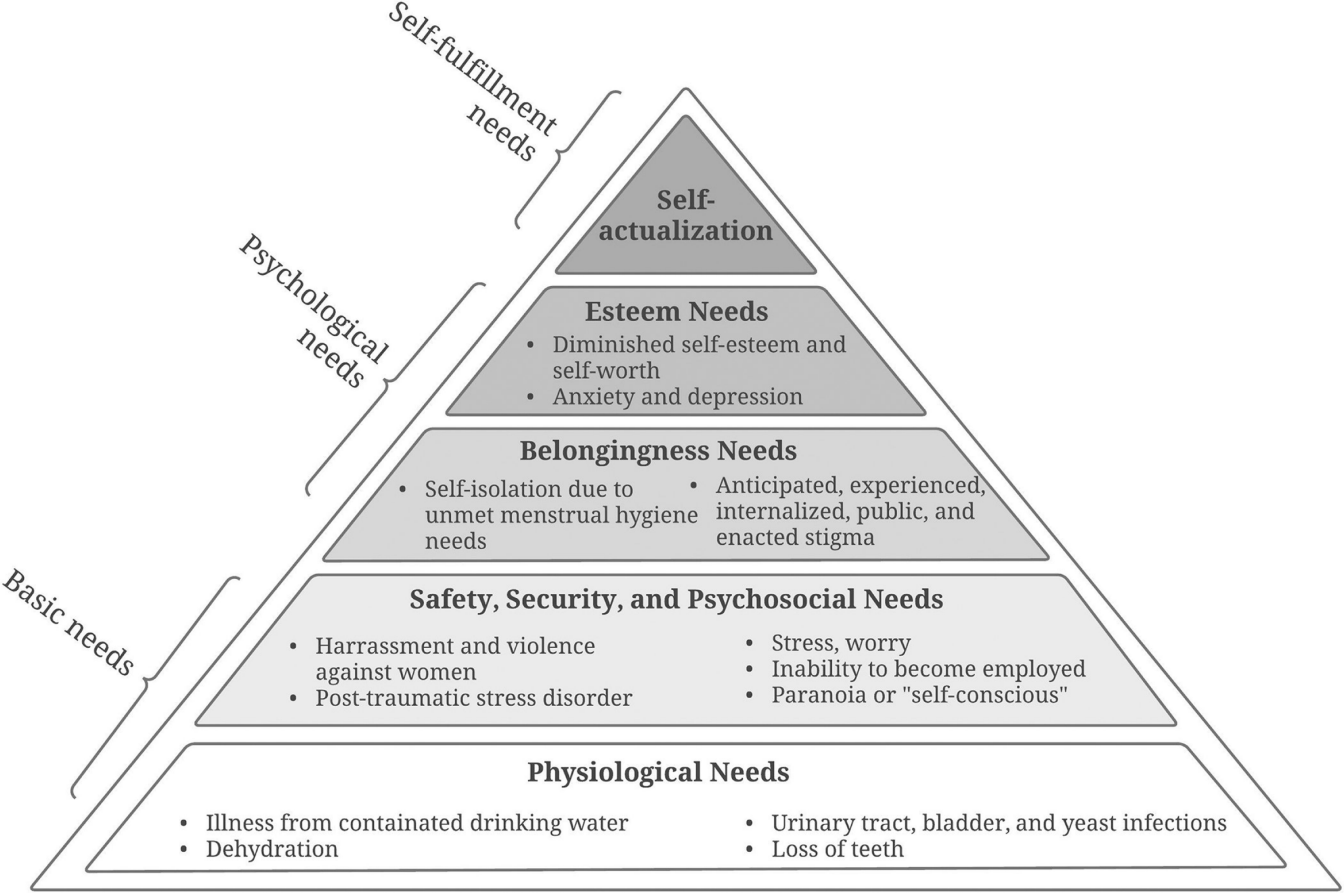
Impacts of inability to access WASH resources and facilities and practice WASH behaviors, aligned with Maslow’s Hierarchy of needs.

Participants’ inability to access WASH resources and facilities and practice WASH behaviors led to adverse basic and psychological health outcomes (Fig 1). Participants’ spent all or most of their time and energy trying to meet basic needs, reducing their mental capacity for securing stable housing and employment.

“Yeah, most people don’t get up every day and worry about how they’re going to eat or where they’re going to clean themselves...Most normal people don’t have to get up and stress about every day basic needs. They’re looking at more of the long-term things like getting jobs and having a car, not where they’re going to get their water or their toilet paper or you know they’re clean clothes.” **(woman, age 35)**

Overall, outcomes among those who currently or previously used drugs were comparable to those who did not. Though, stigma, harassment, and sexual and domestic violence were more prominent throughout homelessness and WASH experiences among those who used drugs.

##### 3.2.1.1. Physiological impacts.

Participants reported adverse WASH-related physical health outcomes including illness from contaminated drinking water; dehydration; urinary, bladder, and yeast infections; and tooth loss due to difficulty caring for their teeth. One participant would not consume water from specific sources due to previous illness, while others continued to drink from “questionable” sources but remained concerned about water quality. A majority experienced dehydration. One participant reported being hospitalized.

“I was in the hospital, I don’t know, a couple of months ago where I lost my bodily fluids because I wasn’t taking enough in. I had to go in and they give me bags of fluids, you know? I feel dehydrated all the time.” **(woman, age 54)**

Female participants reported increased occurrences of urinary, bladder and yeast infections, attributed to their inability to clean their body and clothes and from holding urine for long periods.

##### 3.2.1.2. Safety, security, and psychosocial impacts.

WASH-related impacts on individual safety, security, and psychosocial health were more pervasive than adverse physiological outcomes. Inability to access WASH and practice WASH behaviors caused relentless worry and stress, preventing PEH from meeting higher-level needs such as securing employment. Even if participants were able to consider and search for employment, their unmet hygiene needs would prevent them from becoming employed.

“It even hurts you as far as actually trying to get a job and trying to get back on your feet because people are going to frown on you because you’re not getting to take proper care that you need for yourself...your first impression goes a long way.” **(woman, age 35)**

Women who used drugs anticipated and experienced harassment and sexual and domestic violence; neither men nor women who did not use drugs shared these experiences. To protect themselves from harm, women who used drugs would practice WASH behaviors in private places that did not reveal that they were staying in the area. Harassment and violence were therefore WASH barriers, resulting in some holding urination or defecation when there was no privacy or suitable facility.

The impacts of anticipated and experienced harassment and violence persisted after people were no longer experiencing homelessness and/or using drugs. Some reported symptoms of post-traumatic stress disorder attributed to their homelessness experiences. One woman described feelings of being watched despite being housed at the time of the interview.

“It’s nice to have a toilet and a closed door. It’s made me like an ‘all the time, somebody’s watching me’ feel now because of using the bathroom outside so much...I mean I understand that a door is not a necessity. It’s not all the time going to be there, but it’s nice to have it there so...yeah 100% sure it does [make me feel safer].” **(woman, age 27)**

##### 3.2.1.3. Belongingness impacts.

All participants described adverse impacts of stigma (discussed in section 3.3); however, PEH who used drugs appeared to discuss stigma more often. Those who used drugs experienced intersectional stigma, or the convergence of multiple stigmatized identities within a person, and discrimination based on drug use, personal reputation, and homelessness. Intersectional stigma resulted in feeling ‘othered’ and diminished self-esteem, self-worth, and dignity.

“Everybody looks at you weird because you’re usually wearing the same clothes and people look down on you or whatever so it makes you uncomfortable sometimes...it’s hard...” **(man, age 29)**

Sense of connection was also impacted by self-isolation due to unmet menstrual hygiene needs. Some females reported feeling “disgusting” and “nasty” because of stigma and their menstrual management strategies, like using toilet paper, rags, and socks to manage bleeding or their inability to clean their bodies. As a result, females would seclude themselves and remain where they were sheltering until they could meet their menstrual needs or their period ended.

“[My period] was the time of the month that I would avoid coming to town at all and I would be very paranoid on if I smelled okay...it was just a disgusting time...I didn’t want nobody to be around me. Stayed to myself. Very secluded. And that was hard too.” **(woman, age 27)**

##### 3.2.1.4. Esteem impacts.

Unmet WASH needs contributed to diminished self-esteem, self-worth, anxiety, and depression, becoming an added challenge to the insurmountable circumstances PEH were already living in. Some were actively struggling with such outcomes during interviews.

“Oh lord, [it was] devastating. I used to be the most confident, radiant probably person. You’d walk in a room and I’d light it up...I lost that for a long time. I just shut down, got depressed, got put on medication and everything...I mean, it’s the old saying, which is very true, you feel how you look. So if you feel nasty and dirty, I mean you’re going to feel nasty and dirty.” **(man, age 40)**

#### 3.2.2. WASH barriers.

**Fig 2.**
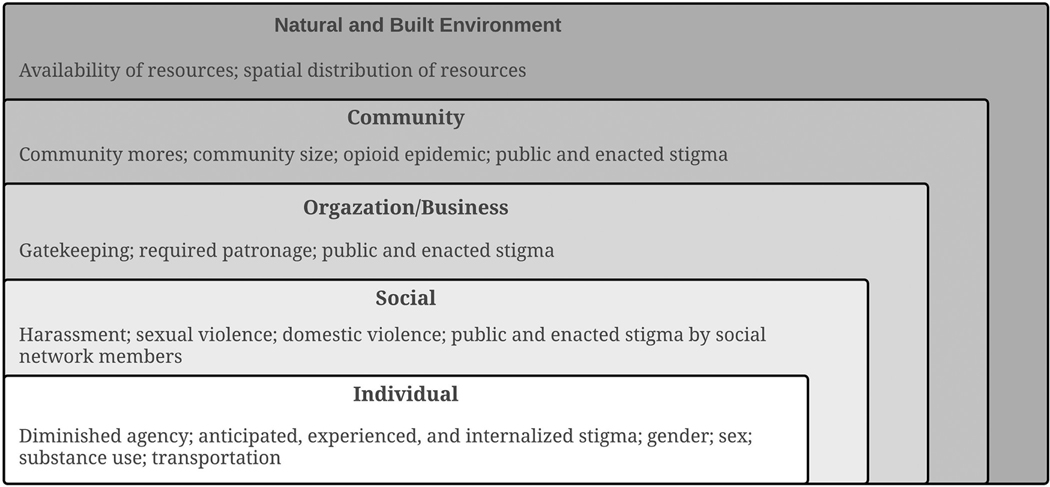
Factors at multiple levels inhibit access to WASH resources and facilities and to performing WASH behaviors among PEH.

Individual agency to access WASH resources and facilities and ability to perform WASH behaviors was diminished for PEH. Factors at multiple levels contributed to diminished agency, serving as WASH barriers, including stigma and place-based characteristics (Fig 2).

##### 3.2.2.1. Stigma.

Stigma at the individual-level was anticipated, experienced, and internalized by participants, and contributed to decreased agency and ability to meet WASH needs and perform WASH behaviors. Those who used drugs uniquely described *intersectional* stigma related to drug use, reputation, and homelessness. Female participants also experienced additional stigma related to menstruation. Shame and disgust led females to seclusion during menstruation, resulting in inability to access resources or facilities.

At the social- and organization/business-levels, stigma was often anticipated or experienced via participants’ social network and during interactions at organizations and/or businesses (i.e., public and enacted stigma). ‘No public bathroom’ signs and procedures, like asking for a key or requiring patronage, were perceived as public stigma by PEH. Employees acted as gate-keepers by limiting or denying access to WASH resources and facilities at organizations and businesses based on physical appearance and/or personal reputation (i.e., enacted stigma).

“They don’t want you to go into the store and use it unless you’re buying something so you go to the porta pottys. So that way you don’t have people looking at you and knowing you’re trying to clean yourself up...They’re putting up signs everywhere ‘no public bath-rooms’...I guess they just don’t want you to go in there and use the restroom.” **(woman, age 54)**

At the community-level, mores related to homelessness influenced participants’ self-esteem and self-worth and compromised WASH access. Those who did and did not use drugs felt that others believed mental illness and substance use were conditional for homelessness. Others made them feel “less than,” “crazy,” and “looked down upon.” Mores surrounding individual or personal responsibility for homelessness and poverty, or the idea that the state of one’s life is a result of one’s own actions and an individual alone can overcome the situational context, made participants feel ashamed of their housing instability. Participants said they “could be doing better” and that “pride” was a WASH barrier. Shame (the opposite of pride) associated with personal responsibility weighed heavily on some, particularly leading those who used drugs to prefer being high when accessing WASH resources.

“[It is hard asking for water at a gas station] sometimes. It’s just one of those, I don’t know, especially if I’m sober, it makes everything a lot more difficult. ‘Cause I guess where I’m high, I don’t really think about it...” **(man, age 29)**

The small size of communities led those who had ever used drugs to experience persistent and prolonged enacted stigma whether they were currently using or not.

“...I used to be a drug addict, but I’ve been clean since September of last year...It’ll follow me around my whole life, I believe, ‘cause nobody can let the past go. If you’ve ever used [drugs], you’re on your own in [study county], I believe. For real.” **(woman, age 27)**

##### 3.2.2.2. Place-based characteristics.

The limited number of resources, spatial distribution of resources, and transportation access impacted WASH access. Participants knew of only a few places where they could meet their WASH needs, and those places had limited capacity or were said to be inaccessible to those with felony charges and/or who use drugs. Additionally, the study area had micropolitan areas (i.e., areas with at least one urban cluster of 10,000–50,000 people), where most businesses and organizations were located. Where PEH stayed or were located directly impacted their WASH access due to this clustering; those who were in more rural parts of the study area struggled more.

“...Everything is clustered together in one spot. So if you’re not downtown or more or less where [restaurant] and things are...then you’re just kind of out of luck.” **(man, age 28)**

However, the type of transportation available to participants (e.g., their own vehicle, walking) influenced how they interacted with their environment.

“...if you have a car, you’re looking at going to a bunch of different restaurants and facilities when you need to use the bathroom. But if you’re looking at not having a vehicle, you’re looking at a lot more in the woods and dealing with buckets and things, trying to keep it confined to an area.” **(woman, age 35)**

## 4. Discussion

We used IDIs to explore WASH experiences and impacts of unmet WASH needs among PEH in Central Appalachian Kentucky, as well as to compare experiences among those who self-reported drug use to those who did not. Our findings revealed that factors at multiple levels, including stigma and place-based characteristics, inhibited WASH access and behaviors at the individual level. Inhibited WASH access and inability to perform WASH behaviors contributed to the adverse physical, mental, and emotional health of PEH. Such impacts exacerbated barriers for PEH, seemingly creating a feedback loop between WASH barriers and adverse health outcomes where inhibited access to WASH resulted in unmet needs and unmet needs inhibited WASH access and behaviors. Though exploratory, our findings highlight the need for place-based and stigma-free approaches that address both the barriers to and impacts of unmet WASH needs for rural PEH. Approaches must address WASH barriers at levels beyond the individual, as demonstrated from our use of the social ecological model, in order to strategically minimize the burden and blame that has historically been placed on PEH (i.e., ideas of self-responsibility) and to effectively develop interventions that account for factors at multiple levels that play a role in behavior and health at the individual level.

Rural PEH face similar and unique challenges to meeting their WASH needs compared to their urban counterparts. Research conducted in urban areas highlights that sanitation and hygiene facilities for PEH are insufficient and/or inadequate due to their limited number, difficulty to access, and lack of privacy and security, among others [11, 43, 48, 49]. Similarly, we found that limited resources and transportation access impacted whether rural PEH could meet their WASH needs. However, rural participants uniquely reported that the clustering of resources and facilities in micropolitan urban cores, a place-based characteristic specific to rural areas, inhibited individuals’ ability to meet their WASH needs.

We found that women faced unique WASH-related challenges and impacts due to menstruation and gender, which is well documented in literature focused on WASH and women, though less so specifically among homeless populations in the US. Similar to findings among women experiencing homelessness in New York City [49] and women throughout varied life stages in rural India [79], rural women experiencing homelessness emphasized the importance of finding safe and private WASH spaces, obtaining period products, and washing their bodies more frequently during menstruation. Rural women secluded themselves during menstruation as seen in Sierra Leone, Bolivia, and the Philippines [80–82]. They also reported having increased occurrences of urinary, bladder, and yeast infections due to inability to clean their bodies and clothes and from holding urine for long periods.

Comparing WASH experiences among PEH who used drugs to those that did not revealed the intricate relationship among WASH, homelessness, and substance use in rural communities that have been impacted by the opioid epidemic. Substance use was described as a catalyst for homelessness, contributing to unmet WASH needs, diminishing self-esteem and self-worth, and leading some to use drugs to cope with the realities of being unhoused. Those who used drugs experienced intersectional stigma related to drug use, reputation, and homelessness, which influenced the type of homelessness experienced and prevented WASH access. Additionally, in our sample, women experiencing homelessness who used drugs uniquely described anticipated and experienced harassment and violence related to WASH that negatively impacted their health and well-being, and served as a WASH barrier while no men or women who did not use drugs shared these experiences. However, other studies in the US have found anticipated and experienced harassment and violence to be common among women experiencing homelessness regardless of drug use [83–85]. This finding may either be unique to rural homeless populations or due to small sample size.

Overall, our findings support the need for place-based approaches that address both WASH barriers and impacts of unmet WASH needs, including strategic placement of stigma-free WASH access points throughout rural communities that are responsive to the unique needs of women. Ideally, public sanitation facilities would provide hygiene and menstrual products for PEH and have shower and laundry access, reducing the burden of meeting WASH needs by centralizing access. Such facilities can and should ideally be expansions to new public WASH infrastructure that has been established during the coronavirus pandemic. For example, the Hand Hygiene for All initiative has prioritized lasting public handwashing infrastructure in response to the coronavirus pandemic and a desire to sustain hand hygiene behaviors [86], which would be crucial to comprehensively meeting PEH’s WASH needs [87]. To prioritize WASH among rural PEH, facilities would need to be spread throughout rural communities rather than clustered in the urban core of micropolitan areas in order to provide access to PEH that lack transportation and/or reside in more remote areas. Access to facilities would need to be available unconditionally (i.e., no ID requirements, no gatekeeping based on felony charges or drug use) to reduce stigma, and be safe and secure to prevent fear of or actual harassment and violence against women.

Collaboration with harm reduction services, which are rapidly expanding in rural areas [88, 89] and provide safe injection equipment to PWID, will be particularly advantageous given that approximately half of rural PEH in this study revealed current or previous drug use, and existing research demonstrates high levels of homelessness among PWUD in areas such as Central Appalachia [65, 66]. Such collaboration could minimize anticipated drug-related stigma and maximize the potential of preventing bacterial injections and abscesses among PEH who inject drugs by facilitating use of clean water to mix or dilute drugs, handwashing, and cleaning the skin prior to injection. Further research is needed to understand the unique drug-related WASH needs for rural PEH who inject drugs, as well as the WASH barriers they experience. Additionally, given the limited amount of research on WASH and rural PEH generally and the small sample size in this study, we emphasize the need for additional qualitative and/or mixed methods research that can facilitate in-depth understanding of the lived experiences and array of adverse health outcomes that rural PEH face both in and outside of the Appalachian context; such work can then serve as the foundation for quantitative research approaches.

## 5. Limitations

Both limitations and strengths of this study should be considered alongside the findings. Use of verbatim transcripts for analyses serves to strengthen the descriptive validity of this study [90]. Additionally, while only one researcher coded the data, debriefing meetings with the data analyst (author AMB) and the senior author (author BAC) were held throughout the analysis process to reduce potential of bias [91]. Conversely, this study was exploratory with a small sample size, which could reduce theoretical validity [90]. Lastly, our comparative analyses by drug use status relied on self-disclosure during IDIs, which may have resulted in misclassification if some participants did not disclose. Nevertheless, comparisons of experiences among PEH who did and did not use drugs is a novel feature of this study given the dearth of research exploring the intersection of rurality, WASH, homelessness, and substance use.

## 6. Conclusion

Our findings underscore homelessness as an emerging public health concern in rural areas such as Central Appalachian Kentucky where rural homelessness is seemingly on the rise, infectious outbreaks are becoming increasingly common (e.g., HAV), and rural PEH face unique WASH-related challenges compared to their urban counterparts. We found substance use to be critical to the discourse on WASH among rural PEH, which seems consistent with recent research showing that homelessness may be prominent particularly among rural PWUD [65, 66]. Place-based approaches that address both WASH barriers and impacts of unmet WASH needs should be prioritized, only made increasingly relevant with rising numbers of homelessness and COVID-19. Expanded WASH facilities that are safe and available to everyone with no prerequisites can address inadequate access among rural PEH and collaboration with harm reduction services may be advantageous to reach those who inject drugs, though additional research will be critical to identifying and implementing specific programs and interventions.

## Supplementary Material

Supplementary material S1 Text. Semi-structured in-depth interview guide.

## Figures and Tables

**Table 1.** Factors that influence types of rural homelessness experienced.

1. Living place-to-place or “couch-surfing”	
Influencing factors:	Example:
• Strong and/or numerous social network ties • Overcoming and leveraging poor social ties and staying somewhere that I s uncomfortable, particularly during winter • Current substance use	“...at the place I’m staying at...[I] walk on eggshells...And uhm, like if I didn’t have there to stay, I don’t know where I would stay...I’m not really welcome there, but [my in-laws] are tolerating me because [my husband] tells them to...It’s hard. It really is.” **(woman, age 27)**
2. Living outdoors or in cars	
Influence factors:	Example:
• Lack of social support • Warm months • Ability to be near natural water resources (e.g., creeks, lakes) and out of sight • Current substance use	“Some people try to camp under the bridge there by the triple creek and that area of the creek helps them a bit...” **(woman, age 35**)
3. Living in structures without water and/or electricity	
Influencing factors:	Example:
• Financial constraints (inability to pay bills) • Lack of infrastructure to access the public water system • Current substance use	“Living in the madness of addiction and stuff, you don’t worry about paying your bills. You know, and even if you do have that worry, you know, that’s the last thing on your mind.” **(woman, age 35)**
4. Living in a shelter for PEH or survivors of domestic violence	
Influencing factors:	Example:
• Knowledge about how shelters function • Requirements to fill out an application, have a photo ID, or no felony charges • Current substance use and policies banning substance use • Lack of privacy and required on-site residency at live-in shelters	“...there are different requirements. Like [the shelter for survivors of domestic violence], I think you got to be in a domestic violence situation and the homeless shelters, they do background checks and you can’t have any, I think you can’t have any felonies or anything like that.” **(woman, age 38)**

**Table 2.** Management strategies among PEH for the various components that constitute hygiene.

Hygiene component	Management strategies	Example
Body	• Bathing or showering in a bathroom at social network members’ houses, at social service organization, while swimming in lakes, or with or without aid of a pan or washcloth in a creek or stream • Using wet wipes, clothing, or paper towels as a washcloth with water and soap to perform “wipe downs,” “freshen up,” or take a “whore’s bath” in a bathroom at a gas stations, convenience store, or restaurant	“Sometimes you use t-shirts for your rags or you might use paper towels in the restrooms that you’re in. Basically use that in substitute for your washcloth. That is something that just has to be thrown away and that goes back to wasting a shirt that could have been worn and that nothing was wrong with it, it’s just all you had available.” **(woman, age 35)**
Hair	• hair in a bathroom at social network members’ houses, in sinks at businesses and restaurants, while swimming in lakes, or with or without aid of a pan in a creek or stream • Using dry shampoo • Cutting or shaving hair off	“I cut my hair off cause I can’t wash my hair all the time, you know?. . . Because you can’t wash your hair all the time so I just shaved the darn thing.” **(woman, age 54)**
Hand and fingernail	• hands in a bathroom at hospitals, businesses, and restaurants, or in water from natural sources (e.g., streams, water puddles) • Using hand sanitizer	“[The] hospital like was amazing...I’d just go over to the bathroom in there and wash my hands in one of the bathrooms in the hospital or in McDonald’s or somewhere.” **(man, age 40)**
Dental	• Brushing teeth in a bathroom at a social network members’ houses, at hospitals, businesses, and restaurants • Using water used for drinking water (e.g., rain water)	“... if you’re camping in the woods, whatever you’d be using for drinking water, you’re still using it to brush your teeth...” **(woman, age 35)**
Clothing	• Using washers and dryers at social network members’ houses or at laundry mats • Using sinks or bathtubs at social network members’ houses, businesses, and restaurants • Using creeks, streams, or water from a shower bag while wearing the clothes and cleaning body, or with or without aid of a scrub board • Throwing items away and purchasing new because it was more affordable	“I would stand by myself and I put my towel up on like two trees or whatever to like make a curtain and I would stand in my clothes though and wash them that way. Then I’d take them off and shake ‘em and rinse ‘em and stuff.” **(woman, age 27)**
Menstruation	• Washing body at social network members’ house • Using wet wipes or soap, and water to wash body or “freshen up” • Using pads, tampons, panty liners, toilet paper, rags, and socks to catch blood	“Tampons. If I didn’t have tampons, I would use toilet paper and there’s only a certain kind of toilet paper you can use...I could make tampons out of toilet paper and I would steal tampons from the store...” **(woman, age 27)**

## Data Availability

Due to the nature of this research (including the sensitivity of topics explored during interviews and criminalization and stigma related to homelessness), participants of this study did not agree for their complete data to be shared publicly, so full interview transcripts are not available. However, participants did agree to de-identified excerpts or quotes from transcripts being available within publications. Thus, supporting excerpts that constitute the minimal dataset have been provided, as relevant, throughout the manuscript. Memos produced during analyses are available upon request as well.
