# Supplementary material for: ‘You feel how you look’: Exploring the impacts of unmet water, sanitation, and hygiene needs among rural people experiencing homelessness and their intersection with drug use
Source: PLOS Water. Author manuscript; Available in PMC 2024 May 13. (PMC11090493; doi:10.1371/journal.pwat.0000019)
Supplement: Supplementary material — S1 Text. Semi-structured in-depth interview guide. [file NIHMS1925935-supplement-Supplementary_material.docx]

INTERVIEW GUIDE

WASH in Rural Areas

Thank you again for taking the time to talk with me today. Again, we are interested in learning about people’s experiences living in this area.

*Open Questions*

1. To get started, please tell me about your experience living in this area.

Probe: What are some of your favorite things about living around here?

Probe: How long have you lived in this area?

Probe: What do you do on a typical day?

*Water*

1. First, I’d like to discuss your experiences with water. Thinking about when you’ve experienced homelessness in this area, describe what a typical day is like for you specifically thinking about water.

Probe: Where do you get water?

-for drinking (e.g., purchasing from a store, water fountains, streams, creeks)?

-for other uses?

Probe: When accessing water during a typical day, what have your experiences been like over time (i.e., similar or different)?

1. Can you tell me about a time when it was difficult for you to get water to drink?

Probe: What about this area or community made it difficult to get water?

-Physical barriers to water sources?

-Social barriers to water sources?

-Variability based on season? Time of day? Need?

Probe: What about this area or community helped you get water?

-Physical access to water sources?

-Social access to water sources (people)?

-Variability based on season? Time of day? Need?

Probe: Given what you have shared, how do these experiences impact your life?

Probe: Do you have concerns about your health?

*Hygiene*

1. We are going to shift to discussing hygiene so things like washing your hands, your body, and your clothes. Please describe what a typical day is like in relation to those when you’ve experienced homelessness in this area.

Probe: Where do you go and what do you need to:

-wash your hands?

-wash your body?

-wash your clothes?

Probe: When washing your hands, your body, and your clothes during a typical day, what have your experiences been like over time (i.e., similar or different)?

1. Please describe a time when you have been unable to find a place to do things such as things like washing your hands, your body, and your clothes.

Probe: What did you do when you needed to wash your hands?

Probe: What did you do when you needed to wash your body?

Probe: What did you do when you needed to wash your clothes?

Probe: What about this area makes it difficult for you to clean yourself and/or your clothes?

-Physical barriers to water sources (soap, shampoo, deodorant, inability to stay

clean?

-Social barriers to water sources (people)?

-Variability based on season (weather)? Time of day? Need?

Probe: What about this area or community helped you clean yourself and/or your clothes?

-Physical access to water sources?

-Social access to water sources (people)?

-Variability based on season? Time of day? Need?

Probe: Given what you have shared, how do these experiences impact your life?

Probe: Do you have concerns about your health?

*Sanitation*

1. Next, we are going to talk about bathrooms. Please describe what a typical day is like specifically thinking about accessing and using bathrooms when you’ve experienced homelessness.

Probe: Where do you use the bathroom?

Probe: When accessing the bathroom during a typical day, what have your experiences been like over time (i.e., similar or different)?

1. Describe a time when you were unable to find and use a bathroom.

Probe: What do you do?

Probe: What aspects about this area made it difficult for you?

-Physical barriers to bathroom?

-Social barriers to bathroom (people)?

-Variability based on season? Time of day? Need?

Probe: What about this area or community helped you access the bathroom?

-Physical access to bathroom?

-Social access to bathroom (people)?

-Variability based on season? Time of day? Need?

Probe: Do these aspects vary based on needs (i.e., urination, defecations, managing menstruation)?

Probe: Given what you have shared, how do these experiences impact your life?

Probe: Do you have concerns about your health?

*Menstruation*

1. You have shared interesting experiences on water, bathing and sanitation. Now, I would like to talk about periods. If you menstruate, what do you do when you get your period?

Probe: What do you use to manage your period (tampons, pads, toilet paper)?

Probe: What do you do when you need to wash your body?

Probe: Given what you have shared, how do these experiences impact your life?

Probe: Do you have concerns about your health?

*Resources*

1. To wrap up, I’d like to talk about resources in the area and recommendations. If you met someone who had recently become homeless, what would you recommend that they do?

Probe: What guidance would you have for them on the topics we discussed today (e.g., water, hygiene, sanitation)?

Probe: What resources would you tell them about?

Probe: What programs would you tell them about?

1. If you were able to create something or do something for the homeless community in this area to help with water, hygiene, and sanitation, what would you do?

Probe: What advice would you give the mayor, government, or someone with a lot of money?

Probe: Related to your other needs (like food, shelter, etc.), would you say this is major, intermediate, or minor in comparison?

Is there anything else you’d like to add or return to before we end the interview?
